# The Association of Congenital Urethral Duplication and Double Megalourethra

**DOI:** 10.4274/balkanmedj.2017.0471

**Published:** 2017-12-01

**Authors:** Murat Uçar, Ahsen Karagözlü Akgül, Nizamettin Kılıç, Emin Balkan

**Affiliations:** 1 Department of Pediatric Surgery, Division of Pediatric Urology, Uludağ University School of Medicine, Bursa, Turkey

**Keywords:** Urethral duplication, urethral anomaly, megalourethra, children

## Abstract

**Background:**

Urethral duplication and megalourethra are rare urethral anomalies. However, the concomitance of urethral duplication and double megalourethra has not been reported previously.

**Case Report:**

A newborn was presented with penile swelling during voiding. Physical examination revealed a retractable foreskin and two external meatus of a double urethra. Retrograde urethrography demonstrated two complete megalourethras. Urethro-urethrostomy and urethroplasty were performed when the patient was 10 months old. The patient was followed up for one year without any urinary problems and has good cosmetics and urinary continence.

**Conclusion:**

The concomitance of these two rare anomalies and more importantly its surgical treatment makes this case report unique and valuable.

Megalourethra is a rare congenital urethral abnormality characterized by dilatation of the anterior urethra without any distal obstruction. Urethral duplication (UD) is a rare congenital malformation that is more frequently found in males and often associated with other abnormalities of the genitourinary tract, heart, bowel and bones. Although both megalourethra and UD may present with associated anomalies, to the best of our knowledge, the concomitance of these two anomalies has not been reported in the English literature previously.

Here, we report a case of type IIA1 complete UD with megalourethra in both urethras. This is the first reported case with the presentation of duplicated megalourethras.

## CASE PRESENTATION

**FIG. 1.**
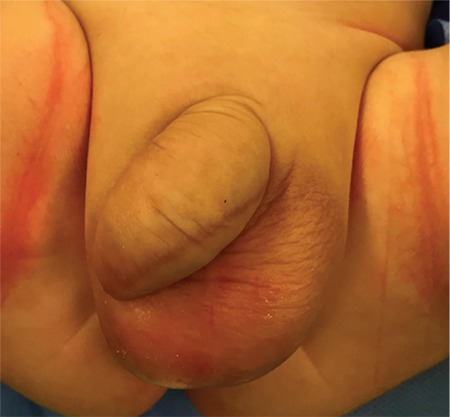
The appearance of the elongated and swollen penis due to megalourethra, preoperatively.

**FIG. 2.**
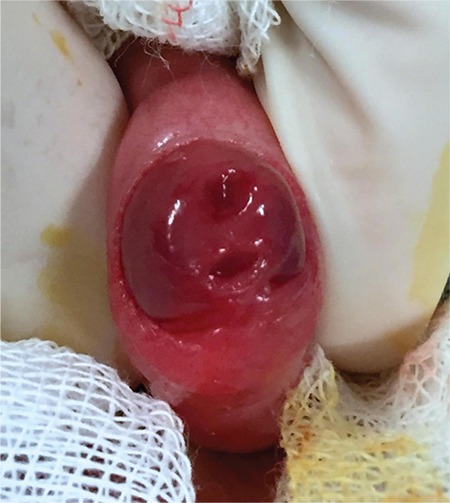
Double urethral meatus on the glans.

**FIG. 3.**
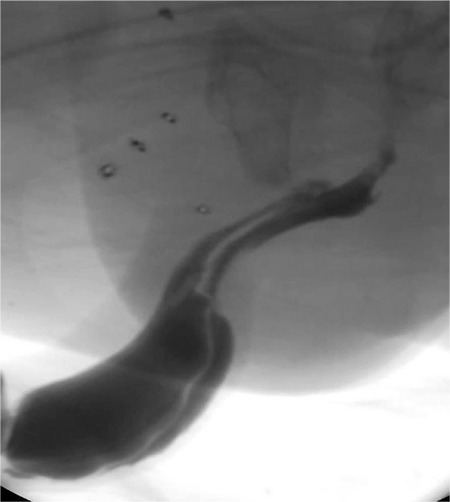
Retrograde urethrography demonstrated the Effmann type IIA2 urethral duplication with megalourethra in both urethras.

A newborn presented with penile swelling (Figure 1). His mother noticed the swelling while he was voiding. Physical examination revealed a retractable foreskin and two external meatus of a double urethra (Figure 2). The dorsal meatus was normally located on the apex of the glans, however the ventral meatus was hypospadiac. There was no history of urinary tract infections. Renal ultrasonography did not reveal any abnormalities in the kidneys. Retrograde urethrography indicated two complete megalourethras (Figure 3).

Urethroscopy was performed via both urethras and determined that while the dorsal urethra opened to the bladder neck with a narrow proximal lumen, the ventral urethra opened to the bladder neck with a normal calibre.

**FIG. 4.**
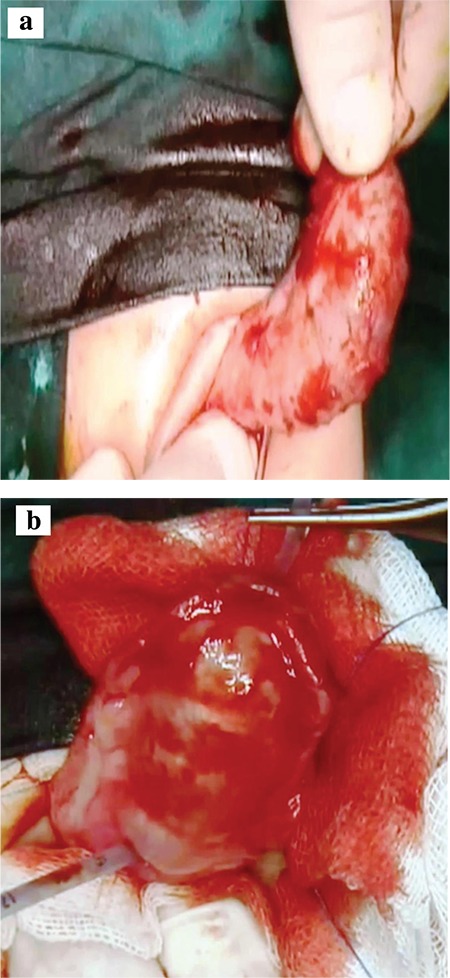
a, b. Dorsal and ventral perioperative views of megalourethras, The ventral megalourethra that was filled with saline to demonstrate the anomaly preoperatively (a), After opening the ventral urethra with a vertical incision, the dorsal megalourethra was shown by filling the urethra with saline (b).

Urethro-urethrostomy and urethroplasty were performed when the patient was 10 months old. During surgery, both urethras were catheterized with 8-Fr catheters and filled with saline to demonstrate the megalourethras (Figure 4a, 4b). Circular incision on the preputial mucosa and degloving were performed. After the ventral and dorsal megalourethras were opened, a 1-cm excision was made in the urethra for tapering of the dorsally placed megalourethra. After excision of the remaining tissues, the common wall of the proximally located normal urethras was repaired. The remaining distally placed urethra was tabularized with two-layer urethroplasty. Glansplasty and penile skin reconstruction were performed at the end of the procedure.

**FIG. 5.**
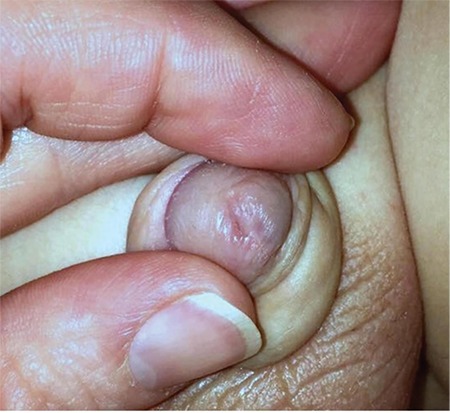
Normal urethral external meatus of the patient in the visit one year postoperatively.

The patient was followed up for one year without any urinary problems and has good cosmetics and urinary continence (Figure 5).

Informed consent was obtained from the parents of the child. Patient-informed consent for publication was obtained after the manuscript was shown to the parents.

## DISCUSSION

Both the UD and the megalourethra are rare urethral anomalies and the concomitance of these two anomalies has not been reported previously. The embryological development of UD is uncertain. There are likely different causes for the various types of anomalies. A misalignment between the termination of the cloacal membrane and its relationship with the developing genital tubercle and urogenital sinus has been reported (1). In 1976, Effmann et al. (2) developed the most widely used classification system for male UD. There are three types in this classification system: type I includes a blind complete UD or accessory urethra, type II includes complete patent UD with four subtypes, and type III includes UD as a component of partial or complete caudal duplication. The duplication in type II may either arise independently from the bladder (type IIA1) or be incomplete, originating from a common proximal urethra (type IIA2), a subtype that includes the ventral urethra terminating in the perineum (Y-duplication) or a rare type in which two urethras arise from the bladder or posterior urethra and terminate in a common channel distally (type IIB) (2).

Megalourethra is a rare congenital urethral anomaly that results from defective development of the corpus spongiosum alone or with hypoplastic corpora cavernosa that occurs in scaphoid or fusiform form, respectively. Megalourethra is more commonly seen in patients with prune belly syndrome and has been reported in association with vertebral defects, anorectal atresia, tracheoesophageal fistula, and renal dysplasia syndrome. The least severe and most common form is the scaphoid variety. The more severe fusiform variety is caused by failure of the penile mesoderm to form spongiosal tissue and corpora cavernosa within the penis.

In most cases, a micturating cystourethrography may be sufficient to demonstrate the anatomical and functional features of the urethras in both anomalies (3). However, in the case of accessory or hyposplastic urethras or non-communication a retrograde urethrography may be required. When planning the surgical procedure, a urethrocystoscopy may be necessary to confirm the diagnosis. In addition, ultrasound scan and/or intravenous pyelography should be used for evaluation of the upper urinary system (4).

Several surgical techniques have been described to treat UD and megalourethra. Treatment of the UD with megalourethra should be individualized according to the anatomy.

In the case of type IIA1 defects, Salle et al. (5) performed either a urethral plate excision or excision of the accessory urethra with or without urethroplasty. The authors also emphasized the importance of identifying the functional urethra and preserving its sphincter mechanism. Performing a complete excision can have a risk of damage to the external sphincter and neurovascular bundle.

In our case, we performed a urethroplasty without excising one of the megalourethras. We were able to create a common distal single urethra with a single meatus, resulting in a good functional urethra and nice cosmetic appearance. The proximal urethras were not dissected and thus we were able to avoid damage to the external sphincter.

To the best of our knowledge, this is the first reported case of a double megalourethra with complete UD. We believe that our successful treatment plan will be a useful addition to the literature and provides a guideline for future cases.
